# Promising CO_2_ gas sensor application of zinc oxide nanomaterials fabricated via HVPG technique

**DOI:** 10.1016/j.heliyon.2024.e36692

**Published:** 2024-08-22

**Authors:** Klaud Jenssen F. Haygood, Dinny Harnany, Jamasri, Gil Nonato C. Santos, Muhammad Akhsin Muflikhun

**Affiliations:** a Physics Department, De La Salle University, Manila, Philippines; b Mechanical Engineering Department, Institut Teknologi Sepuluh Nopember, Surabaya, Indonesia; c Department of Mechanical and Industrial Engineering, Faculty of Engineering, Gadjah Mada University, Yogyakarta, Indonesia

**Keywords:** Zinc oxide rods, The horizontal vapor phase growth, Magnetic field, Root like shape, CO_2_ gas sensor

## Abstract

Highly effective gas sensors for detecting a range of hazardous and toxic gases were successfully applied in the present study using Zinc oxide (ZnO) nanomaterials. In this work, the horizontal vapor phase growth (HVPG) technique was perfectly capable of the synthesis of zinc oxide (ZnO) nanomaterials. The effect of the growth time with different dwell times was discussed by comparing the SEM-EDX analysis and photoluminescence characterization of the samples. Magnetic field (AMF) was also incorporated to determine the effect of AMF on the synthesis of ZnO nanomaterials. The results showed that the ZnO nanorods and root-like shapes are formed with more than 5 μm length and a few nm diameters. The optimum parameter showed the sensors are shiner than the less effective sensor when applied. The introduction of an external magnetic field led to a reduced energy band gap by a maximum of 15 %. The non-AMF band gap energy value is observed to be between 3.51 and 3.58 eV, while the value obtained using AMF is found to be between 2.94 and 3.22 eV. During the CO_2_ gas sensor test, AMF ZnO nanomaterial samples exhibited higher voltage and gradient compared to non-AMF samples.

## Highlights

• Highly effective gas sensors for detecting hazardous and toxic gases was manufactured.
• HVPG technique perfectly capable method for synthesizing ZnO nanomaterials.
• The magnetic field (AMF) influence the nanomaterial's properties.
• ZnO nanorods and root-like shapes have superior gas sensing capabilities.
• The effectiveness of ZnO nanomaterials as gas sensors is successfully tested and sealed.

## Graphical abstract

**Figure:**
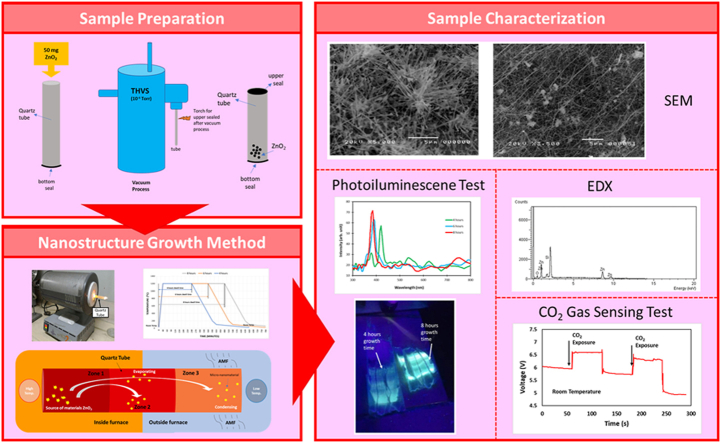

## 1 Introduction

The increasing concentrations of carbon dioxide (CO₂) in the atmosphere have heightened the need for effective and dependable gas detection systems. CO₂ gas sensors are essential for monitoring the environment, ensuring industrial safety, and maintaining healthcare standards. They are critical in detecting and regulating the concentration of this greenhouse gas. The continuous progress in materials science and nanotechnology has greatly accelerated the progress of CO₂ gas sensors, resulting in enhanced sensitivity, selectivity, reaction time, and operational stability [[1], [2], [3]].

Significant advancements have been made in the investigation and application of innovative nanomaterials [4]. Nanostructured metal oxides [5], carbon-based materials [6], and hybrid composites [7] have demonstrated significant promise due to their large surface area, adjustable electrical characteristics, and improved interaction with gas molecules [3]. These materials can be manipulated to demonstrate particular characteristics that improve the ability to capture CO₂ and make it easier to detect even when present in small amounts. Zinc oxide (ZnO) nanoparticles have emerged as a promising candidate for gas sensor applications due to their distinctive chemical and physical characteristics [8,9]. These include a large surface area, superior electrical capabilities, and chemical durability [2,10].

Zinc oxide (ZnO) is a semiconductor with a wide energy bandgap [11,12]. It possesses distinctive attributes, including a large surface area, exceptional chemical stability, and amazing electrical properties [[13], [14], [15]]. ZnO possesses specific characteristics that make it highly appropriate for gas sensing applications. The interaction between gas molecules and the surface of the sensor is essential for optimal performance, and ZnO is well-suited to this task. Nanostructuring can enhance the sensitivity and selectivity of ZnO towards CO₂ by increasing the surface-to-volume ratio and providing more active sites for gas adsorption. The application of ZnO-based CO₂ sensors has been advanced by advancements in sensor design, including the incorporation of ZnO nanoparticles into flexible substrates and the creation of smaller, portable devices [[16], [17], [18], [19], [20]].

The production of ZnO nanostructures represents a crucial field of study, as the qualities and effectiveness of these materials are heavily reliant on their dimensions, morphology, and structural features [[21], [22], [23]]. Zinc oxide (ZnO) nanostructures, such as nanowires [9], nanorods [24], nanoparticles [23,25], and thin films [26], provide a wide range of capabilities, enabling the creation of sensors that may be customized to meet specific sensing needs. A variety of synthesis techniques have been employed to create nanoparticles with precise shape and crystallinity, including sol-gel [27], hydrothermal [28,29], chemical vapor deposition (CVD) [30], and the Horizontal Vapor Phase Growth (HVPG) approach [[31], [32], [33]]. Of these methods, the HVPG technique is notable for its capacity to generate nanostructures of excellent quality, characterized by exceptional uniformity and purity [34]. These attributes are essential for ensuring dependable sensor performance.

The Horizontal Vapor Phase Crystal Growth (HVPG) approach is a method of depositing vapor that involves the spontaneous development of crystals through a process known as vapor-solid (VS) interaction [35]. This technique utilizes the evaporation-condensation process or vapor deposition at an extremely low pressure of 10^−6^ TORR. The HVPG method is based on the alteration of the substance at high temperatures, particularly when it reaches its melting point properties. The process entails the transformation of a solid source material, which has large dimensions, into a liquid condition when it reaches its melting point. This is then followed by evaporation, which is caused by temperature differences in specific areas. As the substance evaporates and comes into contact with a colder area, it condenses and completely sticks to the inner surface of the tube, changing back into a solid state [36].

This study aims to examines the synthesis of ZnO nanomaterials for CO_2_ gas sensors utilizing HVPG, resulting in the formation of ZnO nanomaterials with a highly desirable nano root-like morphology. The impact of growing time and the presence of a magnetic field (AMF) on surface shape, elemental composition, photoluminescence test, and CO_2_ gas sensor performance were investigated. The efficacy of this approach is demonstrated by the successful production of ZnO nanoparticles that can function as CO_2_ gas sensors.

## 2 Materials and method

### 2.1 Materials and specimen preparation

**Fig. 1:**
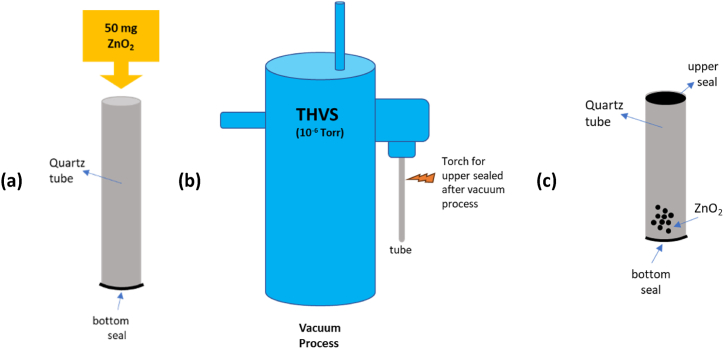
The technique sequences for HVPG and gas sensing test: (a) one end sealed quartz tube, (b) vacuum and sealing process with THVS, and (c) the final substrate before the nanomaterial growth process.

In this investigation, a fused quartz tube with dimensions of approximately 305 mm in length, 11.5 mm in outer diameter, and 8.5 mm in inner diameter was utilized to contain the ZnO_2_ powder. The closure of one end was achieved by employing a high-temperature blowtorch, subsequent to a meticulous 30-min ultrasonic cleaning process. A quantity of 50 mg of ZnO2 powder, sourced from Merck, with a purity of 99 % and a grain size smaller than 5 μm, was carefully inserted into quartz tubes that were clean and well-sealed. The substrate was then carefully placed into the tube. Subsequently, the quartz tube containing the powder was placed vertically in the Thermionic High Vacuum System (THVS) to remove any air and impurities from the tube. The sealing process commences when the pressure reaches 10^−6^ Torr (13.332 × 10^−5^ Pa). The specimen's final length was approximately 150 mm. Fig. 1 depicts the sequential actions involved in preparing the sample.

### 2.2 Fabrication of zinc oxide nanomaterials

**Fig. 2:**
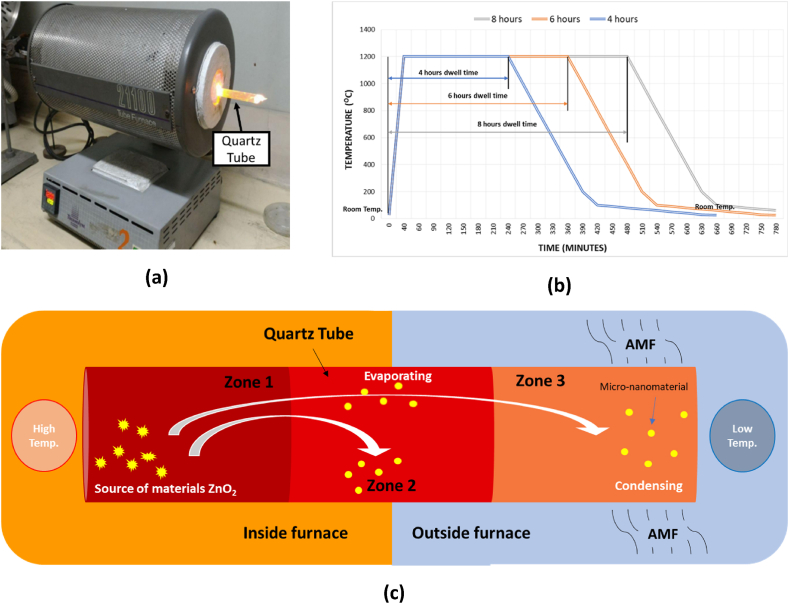
(a) The quartz tube inserted halfway through a horizontal tube furnace, (b) the temperature profile of the furnace during growth the nanomaterial growth process, and (c) the phenomena during the nanomaterial growth process.

During the growth of nanomaterials, the sealed quartz tubes were placed and heated in a Thermolyne horizontal furnace (Fig. 2 (a)). The furnace had a ramp time of 40 min and was set to a specific growth temperature of approximately 1200 °C. The growth process occurred over a period of 4, 6, and 8 h, as shown in Fig. 2 (b). The synthesis of ZnO nanomaterial was carried out under two conditions: one including the introduction of a magnetic field (AMF) and the other without. The 300 Gauss AMF configuration employed a PASCO variable gap neodymium permanent magnet, with a quartz tube positioned between its two cylindrical magnets, each having a diameter of 19 mm (0.75 inch). The quartz tube was divided into three sections, each measuring 50 mm in length. In order to determine the necessary temperature difference for the growth of nanomaterials, the tubes were placed halfway into the furnace. Zone 1, containing the ZnO_2_ powder, was situated within the furnace. Zone 2, positioned in the middle of the furnace, was divided into two halves, with one half inside the inner furnace and the other half inside the outer furnace. Zone 3, situated at the opposite end of zone 1, was entirely outside the furnace. In the configuration of the magnetic field setup, the magnetic field supply was positioned at the terminal of zone 3, as illustrated in Fig. 2 (c). Following the designated baking time, the samples were allowed to naturally cool to ambient temperature. Upon characterization of the ZnO nanomaterial, the cooled quartz tube was sealed with tape and the specific areas were labelled using a marker. The region where the permanent magnetic polish is administered is also indicated. The tubes were fractured mechanically using a bench vice within a fume hood. Fig. 2 illustrates the sequence of material expansion and the transfer of heat through three distinct areas.

### 2.3 Characterization

The characterization of the produced ZnO nanomaterial involved the study of its surface shape, elemental composition, photoluminescence, and its capacity to trap CO_2_ for use as a CO_2_ gas sensor. The surface morphology and elemental composition of the produced ZnO nanomaterial were examined using a JEOL-JSM 5310 Scanning Electron Microscope (SEM) and an Oxford EDX Link Isis System, respectively. The specimens were subjected to a 30-s gold coating process at 50 mA using a JEOL JFC-1200 gold coater in order to improve their conductivity. The photoluminescence spectra of the produced nanomaterials were obtained using an Ocean Optics NIR 256–2.5 spectrometer. The photoluminescence spectra of several samples were analyzed when exposed to UV light stimulation. Only the samples collected from zone 3 were subjected to analysis, as this particular zone exhibits the greatest concentration of cultivated nanostructures and is the location where the external magnetic field was applied. The energy gap (*E*_*g*_) of the generated nanomaterials is determined by analyzing the photoluminescence spectra, using the following equation (1) [37]:

(1) $$E_{g}=\frac{h\;c}{\lambda }$$

where $E_{g}$ is the energy gap, h is the Planck's constant (6.626 × 10^−34^ J), c is the speed of light (2.998 × 10^8^ m/s), and λ is the peak wavelength (m).

**Fig. 3:**
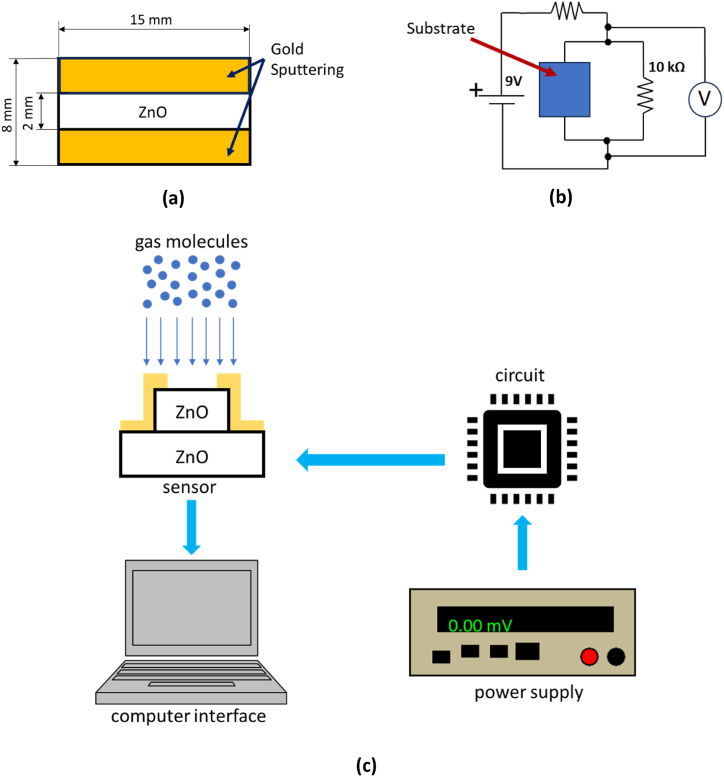
The illustration of (a) ZnO sensor substrate after gold sputtering, (b) circuit diagram of the CO_2_ gas sensor test, (c) flow measurement and data gathering process of the CO_2_ gas sensor test.

The preparation and setup of the CO_2_ gas sensor test are illustrated in Fig. 3. The substrate was subjected to gold sputtering on both sides, which served as electrodes. A 2 mm wide line in the center of the substrate was intentionally left unexposed, as depicted in Fig. 3 (a). The released gas was only identified in nanomaterials that had not undergone gold sputtering. The electrical response of the sensor will be quantified and depicted graphically, illustrating the relationship between voltage and time. To ensure a stable resistance, a 10 kΩ resistor was incorporated into the circuit, as illustrated in Fig. 3 (b). Fig. 3 (c) depicts the application of a Lodestar DC power supply to deliver a consistent input voltage of 9V with a current of 0.5 A. The voltage response was quantified using a Passport Interfaced Pasco Scientific Voltage Sensor, and the data were captured using Data Studio.

## 3 Results and discussion

### 3.1 Surface analysis

**Fig. 4:**
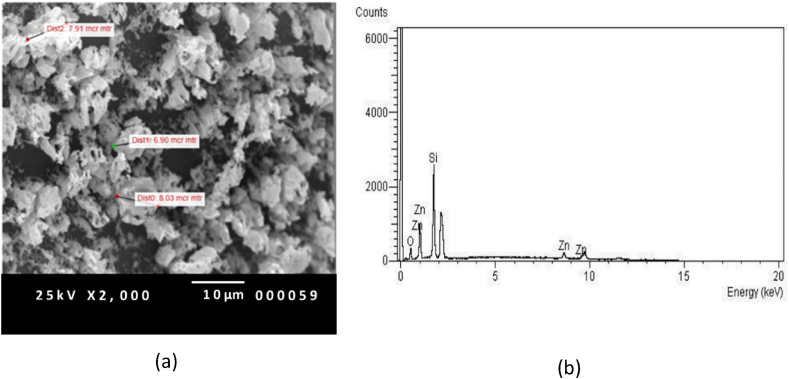
(a) The 2000 magnificent SEM picture and (b) EDX result of ZnO_2_ powder.

The surface morphology and purity of the ZnO₂ powder, which serves as the source material, were examined using scanning electron microscopy (SEM) and energy-dispersive X-ray spectroscopy (EDX), as shown in Fig. 4. The SEM image, magnified 2000 times, reveals that the ZnO₂ powder has a size of roughly 6–8 μm, indicating the absence of a nanostructure. The quality of the source material was confirmed by EDX analysis, which determined an atomic ratio of zinc (64.63 %) to oxygen (35.37 %) that closely approximated the theoretical value of 1:2. It was assumed that the powder was free from any impurities. It can be postulated that the substantial quantity of powdered material initially entrapped within the furnace underwent a transformation from a solid to a gaseous state upon heating, resulting in the formation of nanostructures. This process is believed to have commenced at the furnace's end and beyond its confines. Convection during the heating process caused the vapor within the tube to experience a notable thermal gradient along its length. The hypothesis posits that the source material underwent vaporization within the furnace and was then expelled outside of it. As it moved towards the end of the tube, it reached the cooler area located outside the furnace. At this point, it underwent a change from being a gas to becoming a liquid. Furthermore, it is probable that a portion of the vapor reached the outermost portion of the tube and came into contact with the tube walls in that area, resulting in the condensation of the vapor into liquid due to the dissipation of energy.

**Fig. 5:**
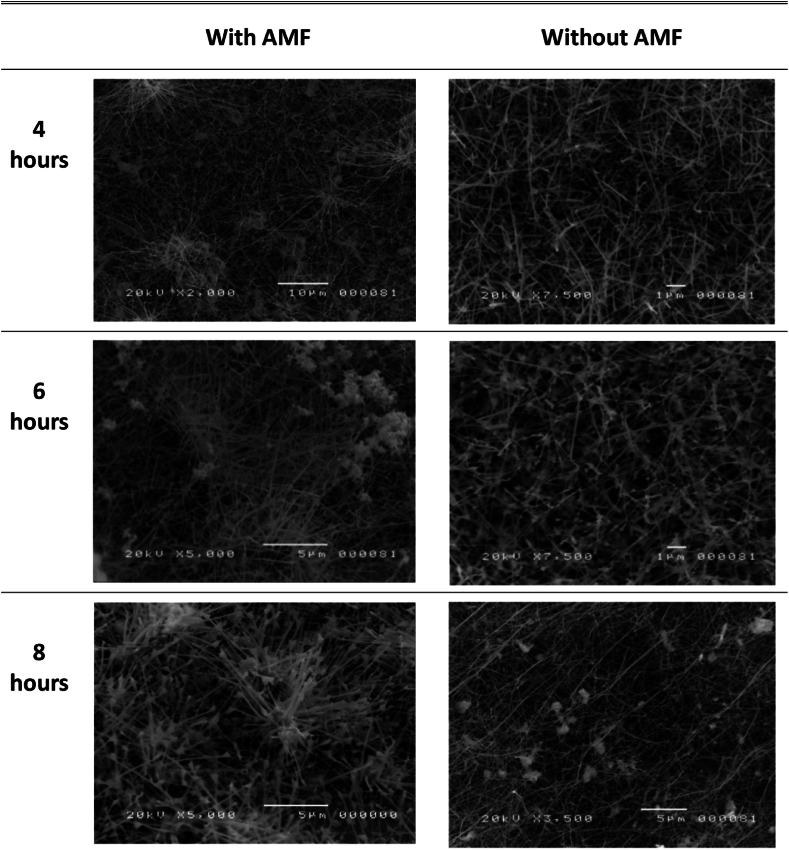
Side by side comparison of ZnO nanomaterial grown in zone 3 (completely outer the furnace).

Fig. 5 presents scanning electron microscopy (SEM) images acquired from zone 3 at different growth durations. There is a positive correlation between the density of nanoparticles and the duration of the process dwell time. Increasing the duration of the stay time results in a higher usage of heat energy from the furnace, which is essential for evaporating the particles. The ZnO_2_ powder was originally positioned in zone 1. The findings indicate that ZnO nanoparticles underwent sublimation and were subsequently transported to cooler areas, specifically zones 2 and 3, while undergoing crystalline growth. The gas convection within the quartz tube is significantly influenced by the temperature difference along its length. It is plausible that some of the vapor was present in the cooler zone during annealing, contributing to the formation of nanomaterials in that particular area. The application of a magnetic field (AMF) during the HVPG process results in the formation of a unique nanostructure. The introduction of a magnetic field causes the nanostructure to display higher density, sharper boundaries, a reduced composition, and a more rigid or linear shape compared to the nanostructure formed without a magnetic field. Magnetic fields can be used to align and organize nanostructures that have magnetic properties or can be magnetized. The magnetic nanoparticles can be directed into specific shapes or patterns by the magnetic field in this state. The findings demonstrated the production of ZnO nanorods with a length exceeding 5 μm and a diameter ranging from 43 nm to 200 nm. The synthesis of ZnO nanorods with a root-like structure and thick regions was successfully achieved by employing an 8-h dwell period, which was determined to be more efficacious than other stay durations.

### 3.2 Elemental composition

**Fig. 6:**
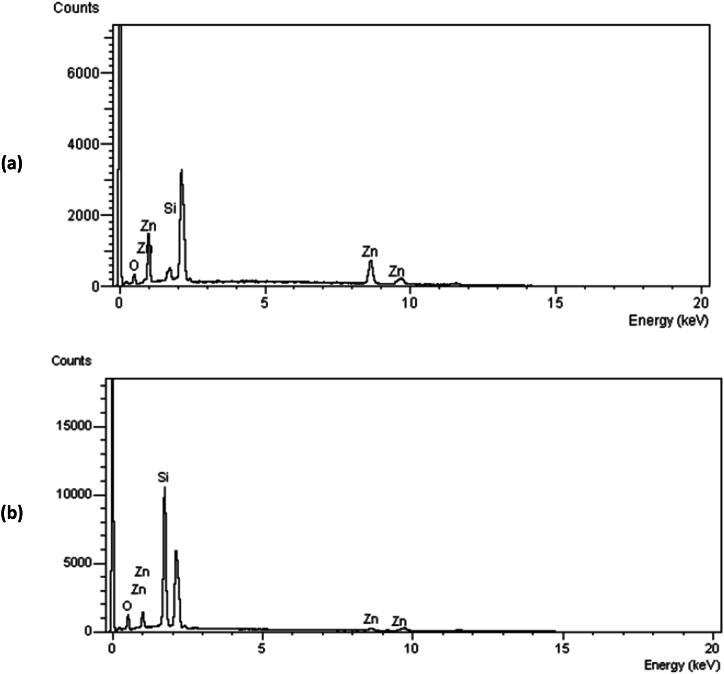
EDX spectrum of the sample synthesized at a growth temperature of 1200^o^C, dwell time of 8 h (a) with AMF and (b) without AMF.

**Fig. 7:**
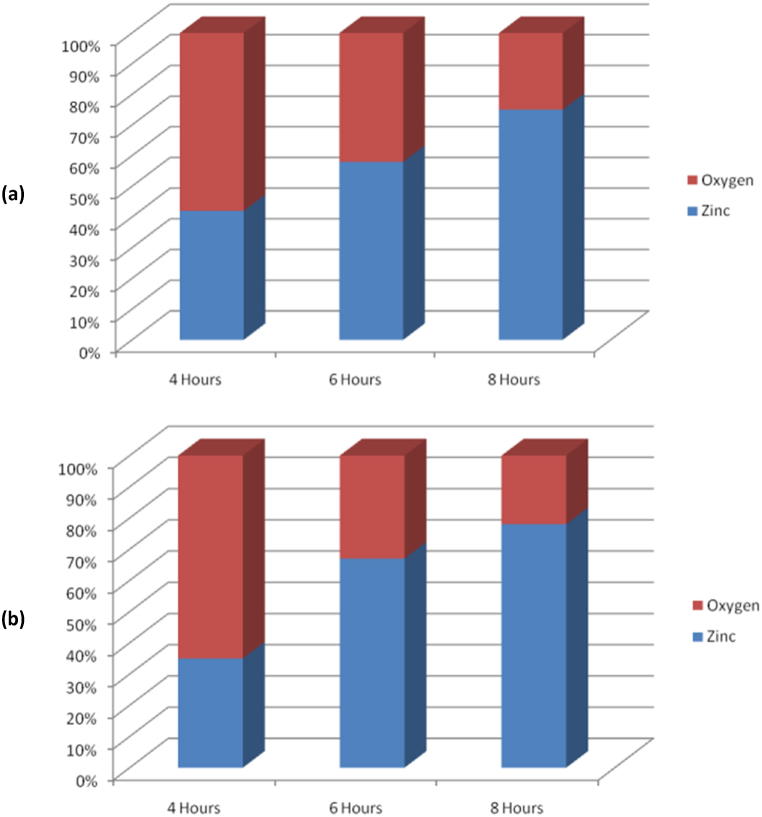
Zn-O atomic percentage of nanomaterials grown (a) with AMF and (b) without AMF.

Fig. 6 presents the results of the EDX spectrum studies conducted on the produced nanomaterial over a period of 8 h. The sample contains ZnO and/or ionized forms of Zn and O, as evidenced by the results. In order to ascertain the precise stoichiometry of ZnO, it is possible to eliminate the influence of quartz by analyzing the relative ratios of Si, O, and Zn. Fig. 7 depicts a notable increase in the number of oxygen vacancies, irrespective of the presence or absence of AMF. The presence of ionized zinc (Zn+) in the nanomaterials is indicated. The data suggest that the ZnO molecule undergoes dissociation into Zn and O at elevated temperatures. The precise ZnO stoichiometry may be uncertain due to unregulated oxidation throughout the growing process.

### 3.3 Photoluminescence (PL) results

**Fig. 8:**
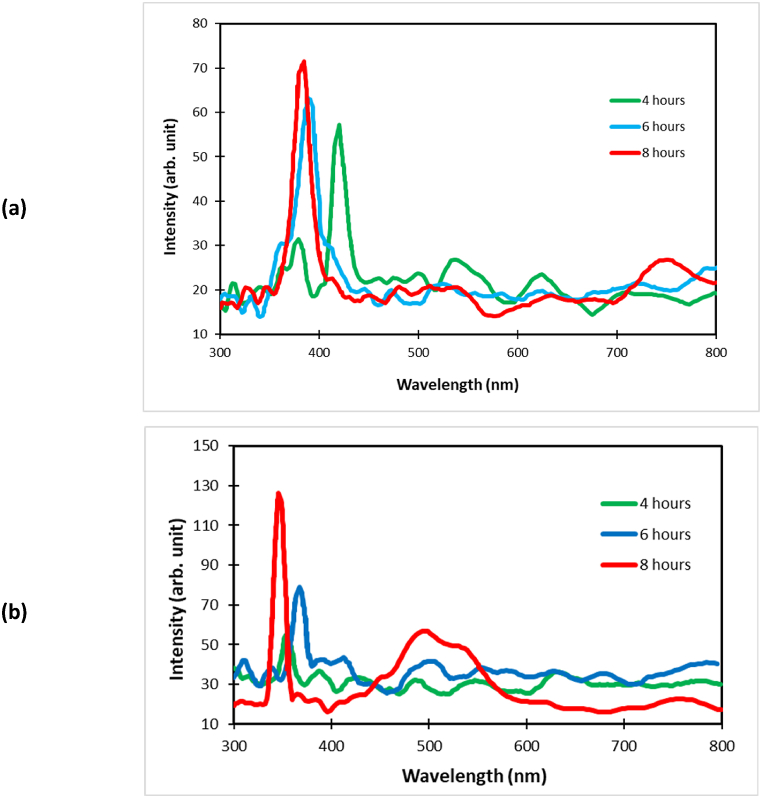
Photoluminescence spectra of the nanomaterials grown at different dwell times (a) with AMF and (b) without AMF

Fig. 8 depicts the photoluminescence (PL) spectra of the nanomaterials synthesized. These spectra were measured at room temperature. The observed ultraviolet (UV) peak is attributed to the recombination of unbound excitons, which is enhanced by an exciton collision event. The band gap energy (e.g.) can be calculated using the following equation (1), with the resulting values presented in Table 1. Samples produced without the application of an AMF exhibited higher band gap energies. Conversely, the application of an AMF during the production of samples results in the formation of more uniform ZnO nanorods, which in turn leads to a reduction in the value of the band gap energy. The most substantial reduction in band gap energy resulting from the application of AMF was observed at a growth time of 4 h, amounting to approximately 15 %. A reduction in the band gap energy is highly advantageous, as it can lead to a decrease in electrical resistance when an electric current is supplied to the specimen. As the duration of the growth period is extended, the corresponding band gap energy value also increases. As illustrated in Fig. 8, the photoluminescence (PL) spectra of the samples that were grown for extended durations exhibited a notable increase in intensity. The higher the intensity, the greater the rate of photon emission during the specified time period.

**Table 1:** Results of the band gap energy calculation.

	Growth Time	Wavelength	Band Energy	
	Hours	m	Joule	eV
**With AMF**	4	4.200 × 10^−7^	4.730 × 10^−19^	2.95
	6	3.881 × 10^−7^	5.118 × 10^−19^	3.19
	8	3.849 × 10^−7^	5.162 × 10^−19^	3.22
**Without AMF**	4	3.530 × 10^−7^	5.628 × 10^−19^	3.51
	6	3.671 × 10^−7^	5.410 × 10^−19^	3.38
	8	3.459 × 10^−7^	5.744 × 10^−19^	3.58

**Fig. 9:**
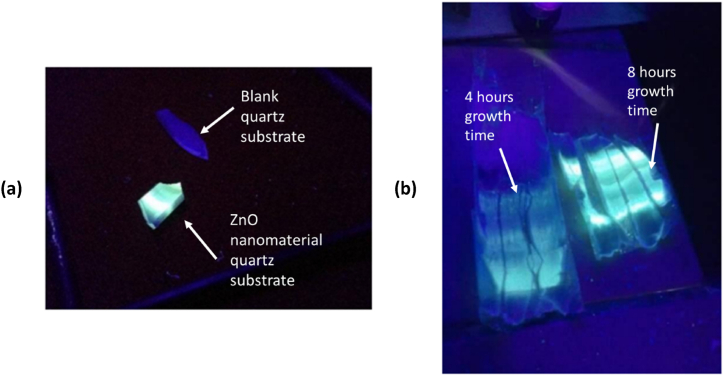
(a) ZnO illuminate under UV light compared to blank quartz substrate, (b) light comparison of 4 h and 8 h growth time quartz substrate under UV light.

Fig. 9 depicts the disparity in the reflection of UV light from the ZnO quartz substrate. Increasing the duration of the development phase leads to a more intense reflection of ultraviolet (UV) light. The shrinking dimensions of the nanostructures resulted in quantum confinement; a phenomenon that occurred due to the extended durations of time required for the production of nanowires with smaller diameters. This led to the emergence of quantum confinement, which increased the band gap of the nanomaterial. Consequently, a shift in the PL spectrum was observed. The visible light spectrum in the longer-grown samples exhibits relatively high intensity peaks, which can be attributed to the presence of a significant number of oxygen vacancies. The presence of oxygen vacancies and a high surface-to-volume ratio contribute to the seemingly unpredictable behavior of the photoluminescence spectra in the visible light range, which is a consequence of the opposing effects of these two factors. The findings demonstrate that the optical properties of the synthesized nanomaterials are markedly influenced by the growth parameters investigated in this study. It can thus be concluded that the optical characteristics of ZnO nanoparticles can be modified by means of controlling the parameters of their growth. Therefore, nanomaterials offer a multitude of applications in optoelectronic devices at the nanoscale.

### 3.4 CO_2_ gas test

**Fig. 10:**
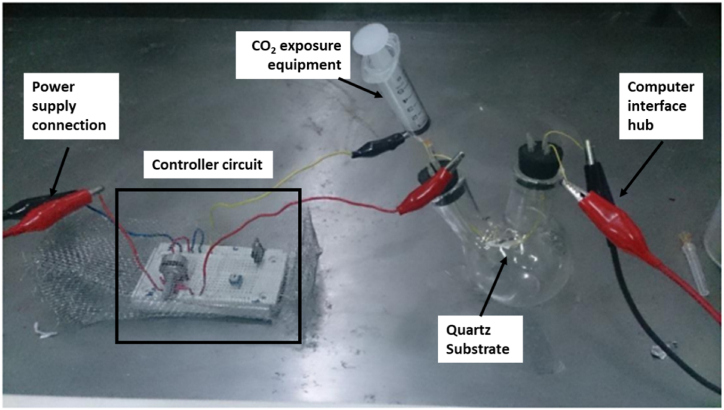
Prototype of the experimental setup.

An evaluation was conducted at room temperature to ascertain the suitability of the produced ZnO nanoparticles for CO₂ gas sensor applications. Fig. 10 depicts the experimental apparatus utilized for testing the CO₂ gas sensor. The quartz tube substrate is positioned within the double-necked glass cup. A syringe is employed to inject CO₂ gas into the glass cup. The experiment was conducted on two substrates: the AMF and non-AMF samples, with a growth duration of 8 h.

**Fig. 11:**
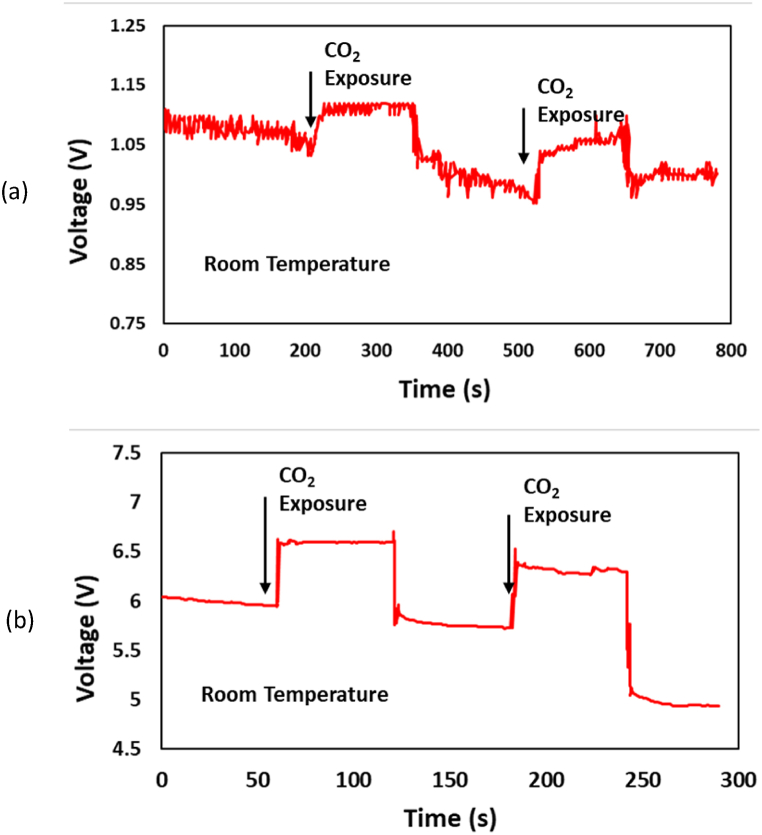
The voltage response of (a) the non-AMF substrate and (b) the AMF substrate sample on CO_2_ exposure.

Fig. 11 depicts the voltage response of both sensor substrates. Both samples exhibited a positive correlation between the voltage reading and the level of CO₂ gas exposure. The presence of CO₂ gas in the sensor substrate functions as an oxidizing agent, causing an increase in the electrical resistance of the substrate and, as a result, the voltage reading. The alteration in resistivity is attributed to the entrapment of electrons by adsorbed molecules and the modification of the energy bands caused by these charged molecules. Furthermore, the presence of adsorbed oxygen species with a negative charge was determined to be the underlying cause of the previously reported phenomenon. The two graphs depict the reaction to an elevation in voltage when carbon dioxide gas is inhaled into a glass cup. However, the AMF sample displayed a more prominent gradient compared to the non-AMF sample. In addition, it was noted that the non-AMF sample generated a more unpredictable electrical output.

The sensitivity of CO_2_ gas sensors is defined as their ability to detect changes in the concentration of CO_2_. This feature is quantified by calculating the ratio of the change in voltage output to the change in CO_2_ concentration. A higher sensitivity implies that the sensor exhibits a more rapid response to variations in voltage. The provided Fig. 11 demonstrates the following: the AMF sensor sample exhibits a fast surge in voltage soon after the release of CO_2_ gas during exhale. Conversely, the non-AMF sensor samples exhibit a more gradual rise in voltage, which occurs over a period of several seconds. The density and arrangement of nanowires on the sample substrate have a significant impact on this phenomenon.

## 4 Conclusions

This study has successfully demonstrated the synthesis of ZnO on a quartz tube using the HVPG method. The scanning electron microscope (SEM) images, in conjunction with energy-dispersive X-ray (EDX) analysis, revealed that nanowires with a diameter of 100 nm or less can only be produced in zone 3 when a 300 G alternating magnetic field (AMF) is employed for a growth period of 8 h in the horizontal vapor phase growth (HVPG) approach. In the absence of an AMF, the sizes of the developed structures were found to be closely correlated with the duration of growth. The presence of an AMF, however, promoted nanowire production, resulting in a more distinct growth pattern and higher density. Furthermore, a higher concentration of nanowire deposition was observed in the specified region when the growth duration was extended to 8 h. The creation of the observed nanowire patterns was attributed to the combined influence of the external magnetic field and temperature differential. Nevertheless, the temperature gradient exerts a more significant impact on the growth of the nanowire compared to the presence of an external magnetic field. The CO_2_ gas detecting capabilities of the ZnO nanomaterial were improved by applying an external magnetic field.

## CRediT authorship contribution statement

**Klaud Jenssen F. Haygood:** Writing – review & editing, Writing – original draft, Visualization, Software, Methodology, Investigation, Formal analysis, Data curation, Conceptualization. **Dinny Harnany:** Writing – review & editing, Writing – original draft, Validation, Software, Methodology, Data curation. **Jamasri:** Writing – review & editing, Writing – original draft. **Gil Nonato C. Santos:** Writing – review & editing, Writing – original draft, Validation, Supervision, Project administration, Funding acquisition, Conceptualization. **Muhammad Akhsin Muflikhun:** Writing – review & editing, Writing – original draft, Validation, Supervision, Project administration, Conceptualization.

## Declaration of competing interest

The authors declare that they have no known competing financial interests or personal relationships that could have appeared to influence the work reported in this paper.
